# A mixed-integer linear programming approach to the reduction of genome-scale metabolic networks

**DOI:** 10.1186/s12859-016-1412-z

**Published:** 2017-01-03

**Authors:** Annika Röhl, Alexander Bockmayr

**Affiliations:** Department of Mathematics and Computer Science, Freie Universität Berlin, Arnimallee 6, Berlin, Germany

**Keywords:** Constraint-based modeling, Model reduction, Stoichiometric models, Mixed-integer linear programming, Metabolic networks

## Abstract

**Background:**

Constraint-based analysis has become a widely used method to study metabolic networks. While some of the associated algorithms can be applied to genome-scale network reconstructions with several thousands of reactions, others are limited to small or medium-sized models. In 2015, Erdrich et al. introduced a method called NetworkReducer, which reduces large metabolic networks to smaller subnetworks, while preserving a set of biological requirements that can be specified by the user. Already in 2001, Burgard et al. developed a mixed-integer linear programming (MILP) approach for computing minimal reaction sets under a given growth requirement.

**Results:**

Here we present an MILP approach for computing minimum subnetworks with the given properties. The minimality (with respect to the number of active reactions) is not guaranteed by NetworkReducer, while the method by Burgard et al. does not allow specifying the different biological requirements. Our procedure is about 5-10 times faster than NetworkReducer and can enumerate all minimum subnetworks in case there exist several ones. This allows identifying common reactions that are present in all subnetworks, and reactions appearing in alternative pathways.

**Conclusions:**

Applying complex analysis methods to genome-scale metabolic networks is often not possible in practice. Thus it may become necessary to reduce the size of the network while keeping important functionalities. We propose a MILP solution to this problem. Compared to previous work, our approach is more efficient and allows computing not only one, but even all minimum subnetworks satisfying the required properties.

**Electronic supplementary material:**

The online version of this article (doi:10.1186/s12859-016-1412-z) contains supplementary material, which is available to authorized users.

## Background

In computational systems biology, genome-scale metabolic network reconstructions are used to build *in silico* models of cellular metabolism [1]. To analyze these models, a large variety of constraint-based methods has been developed over the years [2].

Typically, the metabolic network is assumed to be in steady-state, i.e., the production and consumption of the internal metabolites has to be balanced. This leads to a *flux space* of the form $C = \{ v\in \mathbb {R}^{\text {Rxn}} \mid Sv = 0, \; l \leq v \leq u\}$. Here $S\in \mathbb {R}^{\text {Met} \times \text {Rxn} }$ denotes the *stoichiometric matrix*, given a set of (internal) *metabolites* Met and a set of *reactions* Rxn. The vectors *v*∈*C* are called (feasible) *flux vectors* and can be interpreted as steady-state flux distributions of the metabolic network. The vectors $l,u \in \mathbb {R}^{\text {Rxn}}_{\pm \infty }$ define lower and upper bounds on the fluxes, where $\mathbb {R}_{\pm \infty } := \mathbb {R}\cup \{\pm \infty \}$. By Irrev⊆Rxn we denote the set of *irreversible* reactions, which can carry flux in only one direction, i.e., *v*_*i*_≥0, for all *i*∈Irrev. For simplicity, we assume *l*_*i*_≥0, for all *i*∈Irrev. Reactions in Rev=Rxn∖Irrev are called *reversible*.

Some constraint-based analysis methods can be applied to genome-scale network reconstructions with several thousands of reactions. Others are limited to small or medium-sized models, like the computation of elementary flux modes [3] or minimal cut sets [4]. In such situations, a natural question is whether it is possible to reduce the given large network to a meaningful smaller one of practical size.

In 2015, Erdrich et al. [5] introduced a method called NetworkReducer, which reduces large metabolic networks to smaller subnetworks, while preserving relevant biological properties of interest. The algorithm in [5] is divided into two parts: network pruning and network compressing. In the *compressing* step, reactions belonging to the same enzyme subset [6] are lumped together. In the *pruning* step *removable* and *non-removable* reactions are identified such that the reduced network consisting of the non-removable reactions fulfills four requirements, which can be specified by the user:

1. Set of *protected metabolites* *P* ^Met^: all metabolites in *P* ^Met^ must be retained in the reduced network.
2. Set of *protected reactions* *P* ^Rxns^: all reactions in *P* ^Rxns^ must be retained in the reduced network.
3. Set $\mathcal {F}$ of *protected functionalities* (or phenotypes) for the reduced network. We assume that any protected functionality $f \in \mathcal {F}$ can be described by a corresponding system of linear inequalities: *D*_*f*_ *v*≤*d*_*f*_.
4. Minimum *degrees of freedom*: *dof*≥*dof*_min_. Here, the degrees of freedom *dof* correspond to the dimension of the null space of the stoichiometric matrix *S*, i.e., *dof*=|Rxn|−rank(*S*).

**Fig. 1:**
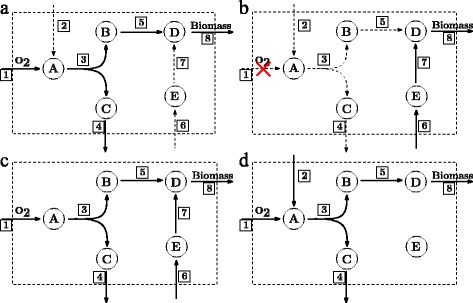
Solid arcs correspond to active reactions, dotted arcs to inactive reactions. In **a**, the flux vector satisfies the functionality of carrying flux through the biomass reaction while having oxygen uptake. In **b**, the functionality is carrying flux through the biomass reaction while there is no oxygen uptake. Combining the two flux vectors leads to the network in **c**, which contains 7 active reactions. A minimum subnetwork enabling both functionalities with only 6 reactions is given in (**d**). The corresponding binary variables for 1d would have the following values: *a*_1_=1,*a*_2_=1,*a*_3_=1,*a*_4_=1,*a*_5_=1,*a*_6_=0,*a*_7_=0,*a*_8_=1, where *a*_*i*_ corresponds to reaction *r*_*i*_

The overall goal of NetworkReducer is to find a smaller subnetwork such that all requirements (1) – (4) can be satisfied by a suitable flux vector. An example is given in Fig. 1.

The method of Erdrich et al. [5] searches for a suitable subnetwork by iterating over the reactions. In every iteration, the flux rate through one particular reaction is set to zero and a linear program (LP) is solved to check if the remaining reactions still form a *feasible* subnetwork. Feasibility means that there exists non-zero flux vectors satisfying the steady-state condition and the other requirements. To identify the reaction to be eliminated a flux variability analysis (FVA) [7] is done and a reaction with smallest overall flux range is selected. Thus in every iteration, an LP is solved and an FVA is performed. Each FVA involves solving up to 2*n* LPs, where *n* is the number of reactions.

**Fig. 2:**
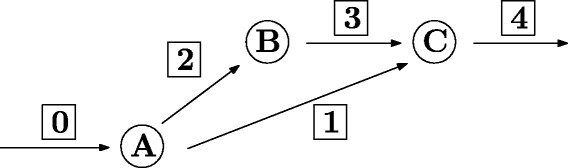
If in the first step of the pruning procedure the flux through reaction 1 is set to zero, reaction 1 is removable and reactions 2 and 3 are non-removable. If in the first step reaction 2 or 3 is set to zero, both of them would be removable and reaction 1 would be non-removable. The resulting subnetwork is then smaller than the first one

An important aspect of the method in [5] is that it does not necessarily compute a minimum subnetwork (with respect to the number of active reactions), see Fig. 2 for an example. The method that we develop here will always find a feasible subnetwork with a minimum number of active reactions. A subnetwork satisfying the requirements (1) – (3) can be obtained by solving only one mixed-integer linear program (MILP). If this subnetwork does not fulfill the *dof*-requirement (4), we exclude this subnetwork and compute a new subnetwork by solving the MILP again. This method turns out to be much faster than the algorithm introduced in [5]. More importantly, we are guaranteed to obtain a minimum subnetwork regarding the number of active reactions, which is not the case for NetworkReducer. However, due to the minimality condition, our method cannot preserve flux variability in the same way as NetworkReducer does.

A second related work is the FASTCORE algorithm of N. Vlassis et al. [8]. This method is also based on solving several LPs but without performing an FVA in between. Thus it is a very fast approach. However, the resulting subnetworks are not minimum and only protected reactions can be specified, but no protected metabolites, functionalities, or degrees of freedom.

An early approach for network reduction was introduced by Burgard et al. [9] already in 2001 and later improved in 2014 by Jonnalagadda and Srinivasan [10]. This method also allows computing minimum subnetworks using an MILP approach. However, only one functionality can be formulated and not several ones like in NetworkReducer. Other related work can be found in [11–18].

Altogether, our method can be seen as a network reduction algorithm that merges features from NetworkReducer and the method in [9], such that we can specify biological requirements like in [5] and compute all minimum subnetworks using an MILP, similar to [9].

The organization of this paper is as follows. In the Methods section we develop the underlying MILP methods. We start with the basic algorithm and then describe several improvements. In the Results and discussion section we compare our MILP approach with the existing methods NetworkReducer and FASTCORE. Furthermore, we apply it to a collection of genome-scale network reconstructions and discuss the results. The last section is Conclusion.

A software tool implementing the algorithms described in this paper is available at https://sourceforge.net/projects/minimalnetwork/.

## Methods

### Basic MILP to compute a minimum subnetwork

We always assume that our network is in steady-state, i.e., *Sv*=0, with bounds on the reaction rates *l*≤*v*≤*u*. Each functionality $f\in \mathcal {F}$ is described by a system of linear inequalities: *D*_*f*_ *v*≤*d*_*f*_. For example, we may require that the biomass reaction has to carry at least 99% of its maximal rate: *v*_Bio_≥0.99· max(*v*_Bio_).

We will use binary variables *a*_*i*_∈{0,1} to indicate whether or not reaction *i* carries flux in the subnetwork. Thus we need the relationship *a*_*i*_=0 if and only if *v*_*i*_=0. For an irreversible reaction *i*∈Irrev, this can be achieved using constraints of the form

1 $$\begin{array}{*{20}l} & \delta \, a_{i} \leq v_{i} \leq Ma_{i}. \end{array} $$

For reversible reactions, we use another binary variable $\bar {a}_{i}$ and the constraints

2 $$\begin{array}{*{20}l} &\delta \, a_{i} - M \, \bar{a}_{i} \leq v_{i} \leq M \, a_{i} - \delta \, \bar{a}_{i}, \quad a_{i} + \bar{a}_{i} \leq 1. \end{array} $$

This type of constraints is called a *big M constraint*, where *M*≫0 is a sufficiently large constant, e.g. some upper bound on the flux rates. With *δ*>0 we denote a threshold indicating above which flux rate a reaction is considered to be active. Practically *δ* will be chosen between 1e^−06^ and 1e^−04^.

To force protected irreversible reactions to carry flux, we use the constraints *a*_*i*_=1 for all *i*∈*P* ^Irr^=*P* ^Rxns^∩Irrev. Enforcing flux through a protected reversible reaction can be realized in a similar way with the constraints $ a_{i} + \bar {a}_{i} = 1,$ for all *i*∈*P* ^Rev^=*P* ^Rxns^∩Rev.

For any protected metabolite *m*∈*P* ^Met^, let Rxn_*m*_ be the set of reactions involving *m*. By Rev_*m*_ we denote the set of reversible reactions in Rxn_*m*_. If Rxn_*m*_ contains at least one protected reaction *r*, metabolite *m* will be protected by reaction *r*. However, if Rxn_*m*_∩*P* ^Rxn^=*∅*, further constraints are needed to protect *m*:

3 $$ \sum_{i \in \text{Rxn}_{m}} a_{i} + \sum_{i \in \text{Rev}_{m}} \bar{a}_{i} \geq 1, \quad \forall m \in {p^{Met}_{0}},  $$

where $p^{Met}_{0} = \left \{ m \in P^{\text {Met}} \mid \text {Rxn}_{m} \cap P^{\text {Rxns}} = \emptyset \right \}$.

In [5], an additional requirement is to specify a minimum number of active reactions. Here we do not include this restriction for the following reasons. First, we will search for the minimum number of active reactions such that all the other requirements are fulfilled. Second, in [5] the minimum number of active reactions is always set to 1. Since there exist reactions which are forced to carry flux, this constraint is redundant.

To find a subnetwork which contains the minimum number of active reactions, we minimize over the sum of the binary variables *a*_*i*_, which indicate whether a reaction carries flux. The resulting MILP is the following:

$$\begin{aligned} & \mathbf{(MinNW-0)}\ \min \sum_{i \in\text{Rxn}} a_{i} + \sum_{k \in\text{Rev}} \bar{a}_{k} \\ & S v = 0, \ l \leq v \leq u & &  \\ & D_{f} v \leq d_{f} & \forall f\in \mathcal{F} &  \\ & \delta \, a_{i} \leq v_{i} \leq Ma_{i} & \forall i \in\text{Irrev} &  \\ &\delta \, a_{i} - M \, \bar{a}_{i} \leq v_{i} \leq M \, a_{i} - \delta \, \bar{a}_{i} & \forall i\in\text{Rev}&  \\ & a_{i} + \bar{a}_{i} \leq 1 & \forall i\in\text{Rev} &  \\ & a_{i} = 1, \enspace a_{k} + \bar{a}_{k} = 1 & \forall i \in P^{\text{Irr}}, \forall k \in P^{\text{Rev}} &  \\ & \sum_{i \in \text{Rxn}_{m}} a_{i} + \sum_{i \in \text{Rev}_{m}} \bar{a}_{i} \geq 1, & \forall m \in {p^{Met}_{0}} & \\ & v_{i} \in \mathbb{R}, \enspace a_{i} \in \{0,1\} & \forall i \in \text{Rxn} & \\ &\bar{a}_{k} \in \{0,1\} & \forall k \in \text{Rev} & \end{aligned} $$

### Conflicting functionalities

In the case study considered in [5], the resulting subnetwork should keep two desired functionalities: under both aerobic and anaerobic conditions at least 99.9*%* of the maximal growth rate should be maintained. These two requirements cannot be realized with the same flux vector *v* because they imply two opposite states of the reaction $\phantom {\dot {i}\!}r_{\mathrm {o}_{2}}$ that transports *O*_2_ into the network. We would need a vector *v* with $\phantom {\dot {i}\!}v_{\mathrm {o}_{2}} \geq \delta $ and $\phantom {\dot {i}\!}v_{\mathrm {o}_{2}} = 0$ at the same time, which is not possible.

MinNW-0 computes one feasible flux vector *v* of the network. But, to get a subnetwork which fulfills the two functionalities we need one flux vector which fulfills the aerobic condition and another one for the anaerobic condition, see Fig. 1. To realize this with a single MILP we have to modify MinNW-0. First, we search for a flux vector *v* ^0^ which contains the protected metabolites and protected reactions. Additionally, for each functionality $j \in \mathcal {F}$ we search for a flux vector *v* ^*j*^ satisfying *D*_*j*_ *v* ^*j*^≤*d*_*j*_ and corresponding binary variables. For example, in Fig. 1, we would have *a*_1_=1 in case 1a) and *a*_1_=0 in case 1b). Due to (1) and (2), this would imply *a*_1_=1 and *a*_1_=0 at the same time, which is not possible. Thus we have to use different binary variables ${a_{i}^{j}}$ for ${v_{i}^{j}}$. With this, the Eqs. (1) and (2) become

4 $$ \begin{aligned} \delta \, {a^{j}_{i}} \leq {v^{j}_{i}} \leq M{a^{j}_{i}} ~~~~~~~~~~~~~~~~~~~~~~~~~~\forall j\in \{ 0, \ldots, |\mathcal{F}|\}, \forall i\in\text{Irrev}, \end{aligned}  $$

5 $$ \begin{aligned} \delta \, {a^{j}_{i}} - M \, \bar{a}^{j}_{i} \leq {v_{i}^{j}} \leq M \, {a^{j}_{i}} - \delta \, \bar{a}^{j}_{i}, ~~\forall j\in \{ 0, \ldots, |\mathcal{F}|\}, \forall i\in\text{Rev}, \end{aligned}  $$

6 $$ \begin{aligned} {a^{j}_{i}} + \bar{a}^{j}_{i} \leq 1 ~~~\forall j\in \{ 0, \ldots, |\mathcal{F}|\}, ~~~\forall i\in\text{Rev}. \end{aligned}  $$

Using the new variables ${a_{i}^{0}}$, we reformulate the constraints regarding the protected reactions: $ {a^{0}_{i}} = 1$, for all *i*∈*P* ^Irr^ and $ {a^{0}_{i}} + \bar {a}^{0}_{i} = 1$, for all *i*∈*P* ^Rev^. Finally, the constraints regarding the protected metabolites become

7 $$ \sum_{i \in \text{Rxn}_{m}} {a^{0}_{i}} + \sum_{i \in \text{Rev}_{m}} \bar{a}^{0}_{i} \geq 1, \quad \forall m \in {p^{Met}_{0}}.  $$

To obtain a minimum subnetwork, we have to minimize the total number of active reactions. Thus, we need binary variables *a*_*i*_ with the property

$$\begin{aligned} a_{i} &= 0~ \text{if and only if}~{a_{i}^{j}}\\ &=0~ \text{for all~} j\in \{ 0, \ldots, |\mathcal{F}|\}, \text{or equivalently}  \\ a_{i} &= 1~ \text{if and only if~} {a_{i}^{j}} =1~ \text{for some~} j\in \{ 0, \ldots, |\mathcal{F}|\}.  \end{aligned} $$

For irreversible reactions, this can be encoded by the constraints

8 $$\begin{array}{*{20}l} a_{i} \enspace \leq \enspace \sum_{j=0}^{|\mathcal{F}|} {a_{i}^{j}} \enspace \leq \enspace (1+|\mathcal{F}|) \cdot a_{i}, \quad \forall i\in\text{Irrev},  \end{array} $$

and for reversible reactions we get

9 $$\begin{array}{*{20}l}{} a_{i} \enspace \leq \enspace \sum_{j=0}^{|\mathcal{F}|} \left({a_{i}^{j}} + \bar{a}_{i}^{j} \right) \enspace \leq \enspace (2 + 2 |\mathcal{F}|) \cdot a_{i}, \quad \forall i\in\text{Rev}.  \end{array} $$

The resulting MILP is the following:

$$\begin{aligned} &\mathbf{(minNW)}\ \min \sum_{i \in\text{Rxn}} a_{i} & \\ & S v^{j} = 0, \enspace l \leq v^{j} \leq u \qquad\qquad\qquad\qquad\qquad~~~~~~ \forall j\in \{ 0, \ldots, |\mathcal{F}|\}& \\ & D_{j} v^{j} \leq d_{j} \qquad\qquad\qquad\qquad\qquad\qquad \qquad~~~~\forall j\in \{ 1, \ldots, |\mathcal{F}|\} & \\ & \delta \, {a^{j}_{i}} \leq {v^{j}_{i}} \leq M{a^{j}_{i}} \qquad\qquad \qquad\qquad~~~~\forall j\in \{ 0, \ldots, |\mathcal{F}|\}, \forall i\in\text{Irrev} &  \\ &\delta \, {a^{j}_{i}} - M \, \bar{a}^{j}_{i} \leq {v_{i}^{j}} \leq M \, {a^{j}_{i}} - \delta \, \bar{a}^{j}_{i} \qquad \qquad~~\forall j\in \{ 0, \ldots, |\mathcal{F}|\}, \forall i\in\text{Rev}&  \\ & {a^{j}_{i}} + \bar{a}^{j}_{i} \leq 1 \qquad \qquad\qquad \qquad\qquad~~~~\forall j\in \{ 0, \ldots, |\mathcal{F}|\}, \forall i\in\text{Rev} &  \\ & {a^{0}_{i}} = 1, \enspace {a^{0}_{k}} + \bar{a}^{0}_{k} = 1 \qquad \qquad\qquad\qquad~~~~~~~~ \forall i \in P^{\text{Irr}}, \forall k \in P^{\text{Rev}} &  \\ & \sum_{i \in \text{Rxn}_{m}} {a^{0}_{i}} + \sum_{i \in \text{Rev}_{m}} \bar{a}^{0}_{i} \geq 1, \qquad\qquad\qquad\qquad\qquad ~~~~~\forall m \in {p^{Met}_{0}} & \\ & a_{i} \enspace \leq \enspace \sum_{j=0}^{|\mathcal{F}|} {a_{i}^{j}} \enspace \leq \enspace (1+|\mathcal{F}|) \cdot a_{i}, \qquad \qquad\qquad\qquad~~~\forall i\in\text{Irrev} &  \\ & a_{i} \enspace \leq \enspace \sum_{j=0}^{|\mathcal{F}|} \left({a_{i}^{j}} + \bar{a}_{i}^{j} \right) \enspace \leq \enspace (2 + 2 |\mathcal{F}|) \cdot a_{i}, \qquad\qquad\qquad \forall i\in\text{Rev} &  \\ & {v_{i}^{j}} \in \mathbb{R}, \enspace {a^{j}_{i}}, a_{i} \in \{0,1\}, \qquad\qquad\qquad~~~~ \forall j\in \{ 0, \ldots, |\mathcal{F}|\}, \forall i \in \text{Rxn} & \\ &\bar{a}^{j}_{k} \in \{0,1\} \qquad\qquad\qquad\qquad\qquad~~~~~ \forall j\in \{ 0, \ldots, |\mathcal{F}|\}, \forall k \in \text{Rev} & \end{aligned} $$

minNW computes a subnetwork with a minimum number of active reactions while satisfying all the requirements.

#### Example for minNW

The network in Fig. 1 a fulfills the functionality regarding the aerobic condition, while the network in Fig. 1 b fulfills the anaerobic condition. The combination of the minimum subnetworks corresponding to each functionality does not lead to a minimum subnetwork for both, see Fig. 1. The minimum subnetwork for this example is given in Fig. 1 d.

### Computing all minimum subnetworks

There are scenarios where we have to compute more than one subnetwork. For instance, consider the case where the minimum *dof* (requirement (4)) is larger than 1. If the subnetwork computed with minNW does not have the required *dof*, we have to compute a different subnetwork. Furthermore, the computed minimum subnetwork need not be unique. Thus there may exist different subnetworks which all fulfill the requirements and have the same number of active reactions. So we may be interested in finding *all* minimum subnetworks. To compute different subnetworks we can use the MILP minNW in an iterative way. Whenever a minimum subnetwork is found, we formulate a constraint which excludes this subnetwork as a feasible solution and solve the (extended) MILP again. For that purpose we formulate the following constraints:

10 $${} \sum\limits_{i \in\text{Rxn}} \left(1-{Z^{k}_{i}}\right) a_{i} + \sum\limits_{i \in\text{Rxn}} {Z^{k}_{i}} (1-a_{i}) \geq 1, \quad k = 1,2,\dots  $$

where ${Z^{k}_{i}} = 1$ if reaction *i* carries flux in the subnetwork which was computed in the *k*-th step, otherwise ${Z^{k}_{i}}=0$. Thus (10) guarantees that at least one inactive reaction will become active, or at least one active reaction will become inactive in the new solution.

Solving minNW iteratively and adding the constraints (10) in each step, we are now able to enumerate all minimum subnetworks.

### Reducing the number of binary variables

To further improve efficiency, we will make use of flux coupling information [19–22]. We first recall some basic definitions from flux coupling analysis (FCA).

A reaction *r*∈Rxn is called *blocked* if *v*_*r*_=0 for all $v \in C_{0} = \left \{ v\in \mathbb {R}^{\text {Rxn}} \mid Sv = 0, v_{i} \geq 0, \forall i \in \text {Irrev} \right \}$. In a pre-processing step, blocked reactions will be removed from the network, which is also done in [5]. Thus we assume from now on that the network contains only unblocked reactions.

Given two unblocked reactions *r,s*∈Rxn, we say *r* is *partially coupled* to *s*, and write *r*⇔*s*, if *v*_*r*_=0⇔*v*_*s*_=0, for all *v*∈*C*_0_. The relation *r*⇔*s* is reflexive, transitive and symmetric and therefore defines an *equivalence relation* on Rxn. This means that the set of reactions Rxn can be partitioned into equivalence classes [*r*]={*s*∈Rxn∣*r*⇔*s* }. It follows $\text {Rxn} = \bigcup _{[r]\in \overline {\text {Rxn}}} \; [r]$, where $\overline {\text {Rxn}}$ denotes the set of all equivalence classes. An equivalence class can be represented by any of its elements. We say that *r* is a *representative* of [*r*] or that [*r*] is the *coupling class* of *r*. Note that [*r*]=[*s*] iff *r*⇔*s*. Biologically, coupling classes can be interpreted as subsets of reactions that are always active together at steady-state, similarly to the notion of enzyme subsets in [6].

The main advantage of introducing coupling classes is that, if one reaction in a class is not carrying flux, no other reaction in the class does, and vice versa. Therefore, in every approach where binary variables are used to indicate if a reaction appears or not, it suffices to consider one reaction from every coupling class instead of considering all of them. Depending on the number of reactions and associated coupling classes, this may significantly reduce the number of required variables.

Based on the equivalence relation *r*⇔*s*, we now use binary variables corresponding to the coupling classes [*r*] instead of having binary variables for each individual reaction. Thus we can rewrite the algorithm minNW in the following way:

$$\begin{aligned} &\mathbf{{(minNW)_{rep}}} \min \sum_{[r] \in \overline{\text{Rxn}}} |\left[r\right]|~ a_{[r]} & \\ & S v^{j} = 0, l \leq v^{j} \leq u \qquad\qquad\qquad\qquad\qquad~~~~~~~~ \forall j\in \{ 0, \ldots, |\mathcal{F}|\} & \\ & D_{j} v^{j} \leq d_{j} \qquad\qquad\qquad\qquad\qquad\qquad\qquad~~~~~ \forall j\in \{ 1, \ldots, |\mathcal{F}|\} & \\ & \delta \, a^{j}_{[r]} \leq {v^{j}_{s}} \leq Ma^{j}_{[r]} \qquad \qquad\quad~~\forall j\in \{ 0, \ldots, |\mathcal{F}|\}, [r]\in \overline{\text{Irrev}}, s\in [r] &  \\ & \delta \, a^{j}_{[r]} - M \, \bar{a}^{j}_{[r]} \leq {v_{s}^{j}} \leq M \, a^{j}_{[r]} \,-\, \delta \, \bar{a}^{j}_{[r]} ~~~\forall j\in \{ 0, \ldots, |\mathcal{F}|\}, [r]\in \overline{\text{Rev}}, s\in [r] &  \\ & a^{j}_{[r]} + \bar{a}^{j}_{[r]}\leq 1 \qquad\qquad\qquad\qquad~~~~~~\forall j\in \{ 0, \ldots, |\mathcal{F}|\}, {[r]}\in\overline{\text{Rev}} &  \\ & a^{0}_{[r]} = 1, a^{0}_{[r']} + \bar{a}^{0}_{[r']} = 1 \qquad\qquad\qquad~~~~ \forall {[r]} \in \overline{P^{\text{Irrev}}}, {[r']} \in \overline{P^{\text{Rev}}} &  \\ & \sum_{[r] \in \overline{\text{Rxn}_{m}}} a^{0}_{[r]} + \sum_{[r] \in \overline{\text{Rev}_{m}}} \bar{a}^{0}_{[r]} \geq 1, \qquad\qquad\qquad\qquad~~~~~\forall m \in \overline{p^{Met}_{0}} & \\ & a_{[r]}\leq \sum_{j=0}^{|\mathcal{F}|} a_{[r]}^{j} \leq (|\mathcal{F}|+1) \cdot a_{[r]} \qquad\qquad\qquad\qquad~~~\forall {[r]}\in\overline{\text{Irrev}} &  \\ &a_{[r]} \leq \sum_{j=0}^{|\mathcal{F}|} \left(a_{[r]}^{j} + \bar{a}_{[r]}^{j} \right) \leq (2+2 |\mathcal{F}|)\cdot a_{[r]} \qquad\qquad~~~~~~\forall {[r]}\in\overline{\text{Rev}} &  \\ &a^{j}_{[r]}, a_{[r]} \in \{0,1\}, {v^{j}_{s}} \in \mathbb{R} \qquad~~~~ \forall j\in \{ 0, \ldots, |\mathcal{F}|\}, \forall [r] \in \overline{\text{Rxn}}, s \in \text{Rxn} & \\ &\bar{a}^{j}_{[r']} \in \{0,1\} \qquad\qquad\qquad\qquad~~~~~~~ \forall j\in \{ 0, \ldots, |\mathcal{F}|\}, \forall [r'] \in \overline{\text{Rev}} & \end{aligned} $$

Here, |[*r*]| denotes the cardinality of the coupling class [*r*]. Thus, we compute the smallest subnetwork with respect to the number of active reactions and not with respect to to the number of active representatives. $\overline {\text {Irrev}}$ denotes the representatives of the irreversible reactions, and $\overline {\text {Rev}} $ those of the reversible reactions. Similarly, $\overline {P^{\text {Irr}}} $ resp. $\overline {P^{\text {Rev}}}$ is the set of representatives of protected irreversible resp. protected reversible reactions. With $\overline {p^{Met}_{0}}$ we denote the representatives which include a protected metabolite.

To exclude previously enumerated subnetworks the constraints (10) can be adapted in the following way:

11 $$ \begin{aligned} \sum_{[ r] \in\overline{\text{Rxn}}}\left(1\,-\,Z^{k}_{[r]}\right) a_{[r]} + \sum_{[r] \in\overline{\text{Rxn}}} Z^{k}_{[r]} \left(1\,-\,a_{[r]}\right) \geq 1, \,\,\,\, k = 1,2,\dots \end{aligned}  $$

Using representatives we need only $|\overline {\text {Rxn}}|$ instead of |Rxn| binary variables. For many genome-wide networks, this reduces the number of 0-1 variables by about 1/2, see the examples in Table 1.

**Table 1:** Number of representatives for different genome-wide metabolic networks (computed with F2FC [20])

Model	Reactions	Unblocked	Coupling classes
*Mus musculus*	3726	2436	1489
*E. coli* iJO1366	2583	2369	1399
*S. Typhimurium* LT2	2545	1620	1047
*S. boydii CDC* 3083-94	2592	1546	1016
*K. pneumoniae* MGH 78578	2262	1223	804
*Y. pestis* CO92	1961	1065	639
*S. cerevisiae* S288c	1577	885	558
*G. metallireducens* GS-15	1285	845	330
*M. tuberculosis* iNJ661	1025	800	412
*B. subtilis* 168	1250	658	342
*P. putida* KT2440	1056	652	282
*C. ljungdahlii DSM* 13528	785	526	215
*H. pylori* iIT341	554	501	209
*M. barkeri str. Fusaro*	690	484	174
*S. aureus* iSB619	743	465	224
*T. maritima* MSB8	652	385	148

## Results and discussion

We implemented our MILPs in MATLAB and used CPLEX [23] as a solver like in [5]. For NetworkReducer resp. FASTCORE we used the implementation provided by the authors of [5] resp. [8]. All computations were done on a desktop machine with two processors Intel(R) Core(TM) i5-2400S, CPU 2.50GHZ, each 1 thread. For algorithm minNW _rep_, we computed the coupling classes for partially coupled reactions using the software F2C2 [20].

**Indicator variables**

We implemented two versions of our algorithms. In one version we used the *big M* constraints from the original MILP formulation in the Methods section. We observed that the solutions are highly dependent on the given tolerances in the MILP solver. To increase numerical stability, we implemented a second version using indicator variables and some other features of CPLEX [23]. The use of indicator variables is straightforward. For example, the *big M* constraint *δ* *a*≤*v*≤*M* *a* is replaced by *a*=0⇒*v*=0,*a*=1⇒*v*≥*δ*, where *a*∈{0,1} is the indicator variable. MILP solvers using indicator variables handle them in two different ways. They may reformulate the given indicator constraints into *big M* constraints or branch on the indicator variables. CPLEX chooses one of these two methods depending on *M*. If *M* is small, it will formulate *big M* constraints, otherwise it will use branching. For the results and the running time we only applied the version where indicator variables were used, due to numerical instability of the *big M* formulation. While indicator variables drastically increase the running time, we still outperform the algorithm in [5].

### Comparison with NetworkReducer

In a first experiment, we ran our implementations on the two metabolic network reconstructions and functionalities considered in [5]. Table 2 shows the running time for calculating a subnetwork with the desired properties.

**Table 2:** Time (in seconds) needed to compute a subnetwork with given requirements resp. constraints

Algorithm	*Synechocystis* sp. PCC 6803		*E. coli* iAF1260	
	Time	reactions	Time	reactions
NetworkReducer	324	462	21987	455
minNW	31	453	4074	416

NetworkReducer: The algorithm introduced in [5]. minNW: The MILP introduced here, using indicator variables

For *Synechocystis* sp. PCC 6803, the subnetwork computed by NetworkReducer [5] contains 462 reactions and thus 9 reactions more than the minimum subnetwork with 453 reactions obtained by our method. The two subnetworks have 413 reactions in common. 49 reactions in the larger subnetwork cannot be found in the minimum subnetwork, while 40 reactions in the minimum subnetwork do not appear in the larger one.

Regarding *E. coli* iAF1260 we get similar results. The subnetwork computed by NetworkReducer contains 39 reactions more than the minimum subnetwork obtained by our method. Both networks have 424 reactions in common. There are 51 reactions that can only be found in the subnetwork computed with NetworkReducer, while there are 12 reactions which appear only in the minimum subnetwork.

### Comparison with FASTCORE

FASTCORE [8] is a heuristic algorithm which is much faster than our method. However, the computed subnetworks are not minimum as can be seen from Table 3. The subnetwork computed with our method is not contained in the subnetwork computed with FASTCORE.

**Table 3:** rxns: number of unblocked reactions in the original network

		rxns	time	rxns	time
Models	rxns	FASTCORE	FASTCORE	minNW	minNW
*M. tuberculosis* iNJ661	1025	134	0.12	62	8727
*H. pylori*	501	319	0.6	306	123
26695					

rxns FASTCORE: number of the reactions in the subnetwork computed with FASTCORE. time FASTCORE: running time in seconds of FASTCORE. rxns minNW: number of the reactions in the subnetwork computed with minNW. time minNW: running time in seconds for the algorithm minNW using indicator variables

For *H. pylori* 26695 there are 22 reactions that appear only in the FASTCORE subnetwork and 9 reactions which can be found only in the minimum subnetwork. Similarly, for *M. tuberculosis* iNJ661, there are 78 reactions that appear only in the FASTCORE subnetwork and 6 reactions which can be found only in our subnetwork. The names of the reactions for both examples are given in the (Additional file 1).

### Network reduction for genome-scale metabolic networks

As a proof of concept we applied our methods to compute minimum subnetworks for 16 metabolic network reconstructions taken from BiGG Models [24] under different scenarios. For each type of organism in BiGG we considered one model (except for human recon because there is no biomass reaction). An overview of the results is given in Table 4. In some cases we had only one minimum subnetwork, while for some models and scenarios we found different ones. For example, in the case of *H. pylori* 26695, we get 16 distinct minimum subnetworks, which will be discussed in the section Case study: *Helicobacter pylori* 26695.

**Table 4:** Computational results using indicator variables

			ess	rxns	mets	reps	time	time	
Models	rxns	mets	rxns	in SNW	in SNW	in SNW	minNW	minNW _rep_	SNWs
*Mus*	2436	1665	247	351	351	241	49085	2949	1
*musculus*									
*E. coli*	2369	1159	363	562	601	262	704	587	1
iJO1366									
*S. Typhimurium*	1620	1098	305	458	455	277	1565	1507	1
LT2									
*S. boydii CDC*	1546	1019	441	445	450	209	15898	463	1
3083-94									
*K. pneumoniae*	1223	830	203	338	340	188	299	194	1
MGH 78578									
*Y. pestis*	1065	761	279	339	339	171	7544	5970	1
CO92									
*S. cerevisiae*	885	639	262	290	289	195	1225	720	2
S288c									
*G. metallireducens*	845	710	544	557	567	153	257	49	1
GS-15									
*M. tuberculosis*	800	580	314	427	425	168	4065	811	1
iNJ661									
*B. subtilis*	658	500	270	296	300	134	16854	10027	1
168									
*P. putida*	652	539	300	344	348	116	3784	827	7
KT2440									
*C. ljungdahlii*	526	448	369	383	389	118	26	7.6	44
*DSM* 13528									
*H. pylori*	501	381	265	321	323	89	9.8	9.8	16
26695									
*M. barkeri str.*	484	417	289	364	369	90	25.38	24.3	20
*Fusaro*									
*S. aureus*	465	387	71	122	127	75	28	27	1
iSB619									
*T. maritima*	385	331	267	282	280	87	14	5.59	28
MSB8									

rxns: number of unblocked reactions in the original network. mets: number of metabolites in the original network after removing dead-end-metabolites. ess rxns: number of essential reactions in the original network. rxns in SNW: number of reactions in the subnetwork. mets in SNW: number of metabolites in the subnetwork. reps in SNW: number of representatives remaining in the subnetwork. time minNW: running time in seconds for the algorithm minNW. time minNW _rep_: running time in seconds for the algorithm minNW _rep_. SNWs: number of minimum subnetworks which exist and fulfill all the requirements. For detecting the running time, only one subnetwork was computed

Following [4], we call a reaction *essential* if after removing this reaction it is no longer possible to achieve at least *p* *%* of the maximal biomass production rate. Like in [4], we choose *p*=20. A minimum subnetwork where it is possible to achieve a maximal biomass rate constitutes a subnetwork where all essential reactions must be active and so all essential reactions have to be included in the subnetwork. We will present the number of essential reactions for the different models to give an idea how many reactions are additionally needed to have a functional minimum subnetwork including all essential reactions.

The scenarios for the different networks and some conclusions are given next, full details can be found in the (Additional file 1). The bounds on the flux rates are those from BiGG Models.

For the networks *Mus musculus*, *E. coli* iJO1366, *S. Typhimurium* LT2, *S. boydii CDC* 3083-94, and *K. pneumoniae* MGH 78578 the requirements are that at least 99.9% of the maximal biomass rates for the aerobic and anaerobic case can be realized by the subnetwork. For *Y. pestis* CO92 the requirements are that at least 99.9% of the maximal growth rate with glycine uptake and the maximal growth rate without glycine uptake can be realized by the subnetwork. For *S. cerevisiae* S288c the maximal biomass rate with and without ethanol exchange has to be realized by the reduced subnetwork. For *G. metallireducens* GS-15, *C. ljungdahlii DSM 13528*, and *T. maritima MSB8* the maximal biomass rate with H_2_O uptake and without H_2_O exchange has to be realized by the reduced subnetwork. For *M. tuberculosis* iNJ661 one requirement is that at least 99.9% of the maximal growth rate can be achieved. Additionally we defined 36 protected reactions. For *B. subtilis* 168 the requirements are that at least 99.9% of the maximal growth rate with hydrogen uptake and the maximal growth rate without hydrogen uptake can be realized by the subnetwork. For *P. putida* KT2440 one requirement is that at least 99.9% of the maximal growth rate can be achieved. Additionally we defined protected reactions to keep the TCA cycle. For *H. pylori* 26695 one requirement is that at least 99.9% of the maximal growth rate can be achieved. Additionally we defined 28 protected reactions. A detailed discussion of this test case will be given in the next subsection. For *M. barkeri str. Fusaro* the requirements are that at least 99.9% of the maximal growth rate with ammonia uptake and the maximal growth rate without ammonia uptake can be realized by the subnetwork. For *S. aureus* N315 at least 99.9% of the maximal biomass rate with glucose uptake and without glucose uptake has to be realized by the subnetwork.

### Case study: *Helicobacter pylori* 26695

In this section we discuss the results for computing several minimum subnetworks for the metabolic network *H. pylori* 26695 using indicator variables. The requirements are the following:

1. There are 28 protected reactions.
2. The maximal biomass yield is 20.2606, and the subnetworks should be able to produce at least 99.9% of this yield.

**Fig. 3:**
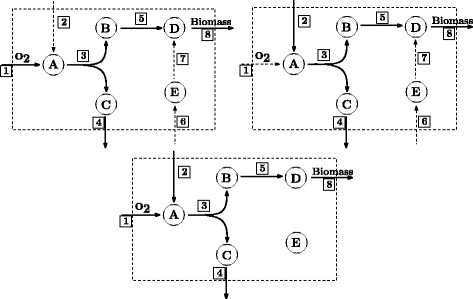
The two illustrations show the distribution of the reactions which are not present in all subnetworks. In Fig. 3 a each reaction (*x*-axis) has a bar. The bar indicates in how many subnetworks the reaction can be found. For example, reaction CCP can be found in every subnetwork except 1 (there are in total 16 subnetworks) and reaction CAT can be found in only one subnetwork. Fig. 3 b illustrates where the reactions are found. Again the *x*-axis corresponds to the reactions. Thus a dot at (1,CCP) means that CCP appears in subnetwork 1. CCP can be found in every subnetwork except in the second one, whereas CAT can be found only in the second one

In total we computed 16 subnetworks each containing 321 reactions, which is the minimum number needed to fulfill the requirements. The time needed to compute all these minimum subnetworks was 127 seconds with minNW and 33 seconds with minNW _rep_. Altogether the 16 minimum subnetworks use 329 different reactions, which can be found in the (Additional file 1). 311 reactions are present in every subnetwork, among them all the 265 essential reactions of *H. pylori*. Only 18 reactions are not present in every subnetwork: CCP, G3PD1, D-Amino acid dehydrogenase, FUMt3, Glycerol-3-phosphate dehydrogenase (NADP), SUCFUMt, L-alanine dehydrogenase, Anthranilate synthase, Formate-tetrahydrofolate ligase, D-Alanine exchange, D-alanine transport via proton symport, L alanine reversible transport via proton symport, L-Alanine exchange, ANS2, GAR transformylase-T, NO3t2, NO3t3, Catalase. Figure 3 shows the distributions of these reactions in the 16 subnetworks.

Additional insight can be obtained by analyzing co-occurrence patterns of the 18 non-essential reactions. Some of these reactions are mutually exclusive regarding the minimum subnetworks. For example, all subnetworks that contain reaction CCP do not contain CAT and vice versa. The same holds for the pair FTHFLi and GART, and the pair ANS and ANS2. Regarding the functionalities of these reaction pairs, one can easily check that the two reactions in each pair do basically the same. Therefore, it is sufficient if only one of them is present. In opposite to this, we can see that DALAt2r and EX_ala__D(e) form a cycle since they always appear in the same subnetworks. The same holds for ALAt2r and EX_ala__L(e). Both cycles also seem to be mutually exclusive, thus only one of them is present in the subnetworks. Similar observations can be made for the cycle formed by NO3t2 and NO3t3, which is mutually exclusive to the cycle formed by SUCFUMt and FUMt3.

One may ask whether the reactions that never appear together in the same subnetwork are also mutually exclusive regarding elementary flux modes (EFMs), i.e., whether or not there exists an EFM involving both reactions [25]. While this holds for the reaction pair FTHFLi and GART and the pair CCP and CAT, it is not true for the other reactions.

## Conclusion

We developed an MILP approach to compute for a given large metabolic network one or more minimum subnetworks preserving biological requirements that can be specified by the user. Compared to previous work [5, 8, 9], our method guarantees minimality of the subnetwork regarding the number of active reactions while preserving all the given requirements. In case there exist several minimum solutions, we are able to enumerate all of them. This may give additional insight how the network is functioning and which reactions are really needed to satisfy the requirements. We applied our algorithms to several genome-scale metabolic networks and we always found all the minimum subnetworks in reasonable time.

Once these subnetworks have been computed, further analysis becomes possible by using methods that are not applicable to the original network. For example, one may compute elementary flux modes and minimal cut sets. In addition, one can take a closer look to the reactions involved in one or all minimum subnetworks in order to get a better understanding of their role in the network.

## Additional file

Additional file 1 Comparison with NetworkReducer, FASTCORE and new experiments. Detailed description of the requirements on the networks and the bounds on the flux rates. (PDF 120 kb)

## Abbreviations

EFM: Elementary flux mode
FVA: Flux variability analysis
FCA: Flux coupling analysis
LP: Linear program/programming
MILP: Mixed integer linear program/programming
