# Supplementary material for: A mixed-integer linear programming approach to the reduction of genome-scale metabolic networks
Source: BMC Bioinformatics. 2017 Jan 3;18:2. doi: 10.1186/s12859-016-1412-z (PMC5210269; doi:10.1186/s12859-016-1412-z)
Supplement: Additional file 1 — Comparison with NetworkReducer, FASTCORE and new experiments. Detailed description of the requirements on the networks and the bounds on the flux rates. (PDF 120 kb) [file 12859_2016_1412_MOESM1_ESM.pdf]

1 Supplement

1.1 Comparison with NetworkReducer

For the reduction of ***Synechocystis* sp. PCC 6803**:

Reactions which were in the subnetwork computed with `NetworkReducer` but not in the subnetwork computed with the MILP-approach `minNW`:

1. PP0007
2. GS0008
3. PP0014
4. PP0009
5. PT0009
6. GS0010
7. GS0004
8. GS0018
9. PP0010
10. PP0013
11. PT0001
12. PP0001
13. TR0050
14. PP0011
15. GE0001
16. PP0012
17. GS0013
18. TR0051
19. GS0014
20. PP0003
21. GS0011
22. TR0052
23. PR0001

24. PDC
25. EtOH_ex
26. Bio_T
27. PR0028
28. PY0016
29. PR0016
30. PR0029
31. PP0020
32. PR0032
33. PR0033
34. PT0026
35. PR0030
36. TE0008
37. PR0031
38. PR0051
39. PT0021
40. PR0004
41. GE0002
42. PR0035
43. PR0049
44. PR0036
45. PR0050
46. TR0036
47. TR0003
48. TR0006
49. TR0002

Reactions which were in the subnetwork computed with the MILP-approach `minNW` but not in the subnetwork computed with `NetworkReducer`:

1. TR0050
2. TR0023
3. GL0003
4. RI0004
5. PU0011
6. PG0008
7. RI0002
8. GE0015
9. CP0007
10. LI0009
11. QT0001
12. TP0002
13. QT0013
14. CA0008
15. LI0058
16. CP0021
17. QT0014
18. AG0020
19. PY0020
20. GL0001
21. LI0004
22. GL0004
23. CA0004
24. PU0010
25. FO0017
26. TR0015

27. LI0033
28. PY0007
29. LI0057
30. GL0005
31. LI0002
32. BM0007
33. GE0004
34. PR0023
35. PR0003
36. PR0027
37. PR0035
38. PR0042
39. TR0055
40. PR0046

For the reduction of ***E. coli* iAF1260**:

Reactions which were in the subnetwork computed with `NetworkReducer` but not in the subnetwork computed with the MILP-approach `minNW`:

1. R_ACALDtex
2. R_ACALDtpp
3. R_ADNK1
4. R_AKGt2rpp
5. R_AKGtex
6. R_CYTBDpp
7. R_D_LACt2pp
8. R_D_LACtex
9. R_EX_acald_e_
10. R_EX_akg_e_
11. R_EX_fe2_e_
12. R_EX_for_e_
13. R_EX_glu_L_e_
14. R_EX_lac_D_e_
15. R_EX_pyr_e_
16. R_FBP
17. R_FE2tex
18. R_FE2tpp
19. R_FORt2pp
20. R_FORtex
21. R_FORtppi
22. R_FRD3
23. R_GART
24. R_GLUN
25. R_GLUSy

26. R_GLUt2rpp
27. R_GLUtex
28. R_GLYCTO4
29. R_GRXR
30. R_GTHOr
31. R_ICL
32. R_LDH_D
33. R_ME1
34. R_ME2
35. R_NADTRHD
36. R_NDPK1
37. R_NDPK6
38. R_PAPSR2
39. R_PPCK
40. R_PPM
41. R_PPS
42. R_PUNP1
43. R_PYRt2rpp
44. R_PYRtex
45. R_SUCCt2_2pp
46. R_THRt2rpp
47. R_THRt4pp
48. R_TRPS2
49. R_TRPS3
50. R_URIDK2r
51. R_RFD_new

Reactions which were in the subnetwork computed with the MILP-approach `minNW` but not in the subnetwork computed with `NetworkReducer`:

1. R_ACOTA
2. R_ADK1
3. R_ADNK1
4. R_CYTBDpp
5. R_DPR
6. R_EX_mn2_e_
7. R_EX_o2_e_
8. R_GAPD
9. R_GARFT
10. R_MTHFD
11. R_RNTR3c
12. R_RNTR4c

1.2 Comparison with FASTCORE

For the reduction of ***M. tuberculosis*** **iNJ661** :

Reaction which were in the subnetwork computed with `FASTCORE` but not in the subnetwork computed withthe MILP-approach `minNW`:

1. TALA
2. SUCCt2r
3. RPI
4. RPE
5. PSP_L
6. PSERT
7. PRPPS
8. PREPTTA
9. PREPTHS
10. PPAtr
11. PHTHS
12. PHTHDLS
13. PGCD
14. PDIMAT
15. PDIMAS
16. MMM2
17. MME
18. MMCD
19. METS
20. METAT
21. UMPK_copy2
22. TKT2
23. TKT1
24. MALS

25. L_LACD3
26. ICL
27. H2Ot
28. GLYCL
29. GLUDxi
30. GLNS
31. GHMT2r
32. FRDO3r
33. FRDO2r
34. EX_h_e
35. EX_h2o_e
36. EX_cytd_e
37. ORPT
38. OMPDC
39. OCOAT1
40. NTPP4
41. NTD4
42. NH4t
43. NDPK1
44. MTHFR2
45. DHORTS
46. CYTDt2
47. CYTBD2
48. CTPS1
49. CITL_copy1
50. CBPS
51. ASPTA
52. ASPCT

53. ARGSS
54. ARGSL
55. ARGDr
56. ARACHTA
57. AHCi
58. ADNK1
59. FASm280
60. FASm260
61. FASm240
62. FASm220
63. FAS80_L
64. FAS200
65. FAS180
66. FAS160
67. FAS140
68. FAS120
69. FAS100
70. FACOALPREPH
71. FACOAL200
72. EX_succ_e
73. EX_ppa_e
74. EX_pdima_e
75. EX_nh4_e
76. ADK1
77. ACCOAC
78. ACACCT

Reactions which were in the subnetwork computed with the MILP-approach `minNW` but not in the subnetwork computed with `FASTCORE`:

1. TALA
2. PFK
3. MALS
4. L_LACD3
5. GLYCL
6. GLUDxi

For the reduction of ***H. pylori* 26695**:

Reactions which were in the subnetwork computed with `FASTCORE` but not in the subnetwork computed with the MILP-approach `minNW`:

1. ACt2r
2. ALAt2r
3. ANS2
4. APRAUR
5. CO2t
6. DAAD
7. DB4PS
8. DHPPDA
9. EX_ac(e)
10. EX_ala__L(e)
11. EX_co2(e)
12. EX_h2co3(e)
13. EX_urea(e)
14. GTPCII
15. H2CO3D
16. H2CO3TP
17. PMDPHT
18. RBFSa
19. RBFSb
20. RNDR4
21. UREAt
22. URIDK2r

Reactions which were in the subnetwork computed with the MILP-approach `minNW` but not in the subnetwork computed with `FASTCORE`:

1. ACt2r
2. ALAt2r
3. AMAOTr
4. AOXSr
5. DASYN_HP
6. DPCOAK
7. EX_ala__D(e)
8. FLDO
9. UAGDP

1.3 New experiments

Mus musculus: The maximal biomass rate with oxygen uptake is 1.3634 h^{-1}. Without oxygen, the rate is 0.0971 h^{-1}. Per default the lower bound of oxygen is -100 mmol/gDW/h and -1 mmol/gDW/h of glucose. The upper bounds of both are 100000 mmol/gDW/h. The requirements are that at least 99.9 % of both rates can be realized by the subnetwork. The original network consists of 2436 non-blocked reactions. All the requirements can be fulfilled by using 338 reactions. The number of essential reactions for having at least 20% of the biomass reaction is 274.

***E. coli* iJO1366**: The requirements are that at least 99.9 % of the maximal growth rate under aerobic conditions (37.9623 h^{-1}) and at least 99.9 % of the maximal growth rate under anaerobic conditions (28.6643 h^{-1}) can be realized by the subnetwork. The original network consists of 2369 non-blocked reactions. Per default the lower bounds of oxygen and glucose are both -1000 mmol/gDW/h. The upper bounds of both are 1000 mmol/gDW/h. All the requirements can be fulfilled by using 562 reactions. The number of essential reactions for having at least 20% of the biomass reaction is 363.

***S. Typhimurium* LT2**: The requirements are that at least 99.9 % of the maximal growth rate under aerobic conditions (34.4108 h^{-1}) and under anaerobic conditions (18.3065 h^{-1}) can be realized by the subnetwork. The original network consists of 1620 non-blocked reactions. Per default the lower bounds of oxygen and glucose are both -1000 mmol/gDW/h. The upper bounds of both are 1000 mmol/gDW/h. All the requirements can be fulfilled by using 458 reactions. The number of essential reactions for having at least 20% of the biomass reaction is 305.

***S. boydii CDC* 3083-94**: The requirements are that at least 99.9 % of the maximal growth rate under aerobic conditions (0.9828 h^{-1}) and at least 99.9 % of the maximal growth rate under anaerobic conditions (0.2418 h^{-1}) can be realized by the subnetwork. The original network consists of 1546 non-blocked reactions. Per default the lower bound of oxygen is -1000 mmol/gDW/h and the one of glucose is -10 mmol/gDW/h. The upper bounds of both are 1000 mmol/gDW/h. All the requirements can be fulfilled by using 445 reactions. The number of essential reactions for having at least 20% of the biomass reaction is 441.

***K. pneumoniae* MGH 78578**: The requirements are that at least 99.9 % of the maximal growth rate under aerobic conditions (32.3593 h^{-1}) and under anaerobic conditions (16.8005 h^{-1}) can be realized by the subnetwork. The original network consists of 1223 non-blocked reactions. Per default the lower bounds of oxygen and glucose are both -1000 mmol/gDW/h. The upper bounds of both are 1000 mmol/gDW/h too. All the requirements can be fulfilled by using 338 reactions. The number of essential reactions for having at least 20%

of the biomass reaction is 203.

***Y. pestis* CO92**: The maximal biomass rate with glycine uptake is 0.2836 h^{-1}. Without glycine, the rate is 0.0886 h^{-1}. Per default the lower bound of oxygen is -20 mmol/gDW/h, -2 mmol/gDW/h of glucose and -2.2 mmol/gDW/h of glycine. The upper bounds of oxygen and glucose are 100000 mmol/gDW/h, the one for glycine is 1000 mmol/gDW/h. The requirements are that at least 99.9 % of both rates can be realized by the subnetwork. The original network consists of 1065 non-blocked reactions. All the requirements can be fulfilled by using 339 reactions. The number of essential reactions for having at least 20% of the biomass reaction is 279.

***S. cerevisiae* S288c**: The maximal biomass rate with ethanol exchange is 0.2879 h^{-1}. Without ethanol, the rate is 0.2311 h^{-1}. Per default the lower bound of oxygen is -2 mmol/gDW/h, -10 mmol/gDW/h of glucose and 0 mmol/gDW/h of ethanol. The upper bounds of all three reactions are 9999999 mmol/gDW/h. The requirements are that at least 99.9 % of both rates can be realized by the subnetwork. The original network consists of 885 non-blocked reactions. All the requirements can be fulfilled by using 290 reactions. The number of essential reactions for having at least 20% of the biomass reaction is 262.

***G. metallireducens* GS-15**: The maximal biomass rate with H_2O uptake is 5.8178 h^{-1}. Without H_2O, the rate is 2.2028 h^{-1}. Per default the lower bound of H_2O is -1000 mmol/gDW/h and of glucose 0. The upper bounds of both are 1000 mmol/gDW/h. The requirements are that at least 99.9 % of both rates can be realized by the subnetwork. The original network consists of 710 non-blocked reactions. All the requirements can be fulfilled by using 557 reactions. The number of essential reactions for having at least 20% of the biomass reaction is 544.

***M. tuberculosis* iNJ661**: One requirement is that at least 99.9 % of the maximal growth rate (0.0525 h^{-1}) can be achieved. Additionally we defined 36 protected reactions. The original network consists of 1025 non-blocked reactions. Per default the lower bound of oxygen and glucose are both -1 mmol/gDW/h. The upper bounds of both are 999999 mmol/gDW/h. All the requirements can be fulfilled by using 427 reactions. The number of essential reactions for having at least 20% of the biomass reaction is 314.
Protected reactions for the network ***M. tuberculosis* iNJ661**:

1. Succinyl-CoA synthetase (ADP-forming)
2. Succinate dehydrogenase
3. Pyruvate decarboxylase
4. Pyruvate kinase

5. PPGKr
6. Propanoyl-CoA: succinate CoA-transferase
7. Phosphoenolpyruvate carboxykinase
8. Phosphoglucomutase
9. Phosphoglycerate mutase
10. Phosphoglycerate kinase
11. Glucose-6-phosphate isomerase
12. Phosphofructokinase
13. PEPCK re
14. PDHbr
15. PDHa
16. Pyruvate dehydrogenase
17. Pyruvate carboxylase
18. Malate dehydrogenase
19. Triose-phosphate isomerase
20. L-lactate dehydrogenase
21. Isocitrate dehydrogenase (NADP)
22. Hexokinase (D-glucose:ATP)
23. Glycogen synthase (ADPGlc)
24. Glyceraldehyde-3-phosphate dehydrogenase
25. Fumarase
26. OXGDC
27. Enolase
28. Citrate synthase
29. Aldehyde dehydrogenase (acetaldehyde
30. NAD)
31. FRD5
32. FRD

33. Fructose-bisphosphatase

34. Fructose-bisphosphate aldolase

35. Acetyl-CoA synthetase

36. Aconitase

***B. subtilis* 168**: The maximal biomass rate with hydrogen uptake is 0.1247 h^{-1}. Without hydrogen, the rate is 0.1186 h^{-1}. Per default the lower bounds of oxygen and hydrogen are -9999999 mmol/gDW/h and of glucose -1.7 mmol/gDW/h. The upper bound of oxygen is 0 and the one of glucose and hydrogen are 9999999 mmol/gDW/h. The requirements are that at least 99.9 % of both rates can be realized by the subnetwork. The original network consists of 658 non-blocked reactions. All the requirements can be fulfilled by using 296 reactions. The number of essential reactions for having at least 20% of the biomass reaction is 270.

***P. putida* KT2440**: One requirement is that at least 99.9 % of the maximal growth rate (1.4000 h^{-1}) can be achieved. Additionally we defined protected reactions to keep the TCA cycle. Per default the lower bounds of oxygen and glucose are both -20 mmol/gDW/h. The upper bounds are 9999999 mmol/gDW/h. The original network consists of 652 non-blocked reactions. All the requirements can be fulfilled by using 344 reactions. The number of essential reactions for having at least 20% of the biomass reaction is 300. Protected reactions for the network ***P. putida* KT2440**:

1. Acetate kinase

2. Aconitase

3. Aconitase (half-reaction A, Citrate hydro-lyase)

4. Aconitase (half-reaction B, Isocitrate hydro-lyase)

5. Oxoglutarate dehydrogenase (lipoamide)

6. Oxoglutarate dehydrogenase (dihydrolipoamide S-succinyltransferase)

7. Citrate synthase

C. ljungdahlii DSM 13528: The maximal biomass rate with H_2O uptake is 0.2245 h^{-1}. Without H_2O, the rate is 0.2027 h^{-1}. Per default the lower bound of H_2O is -1000 mmol/gDW/h and of glucose 0. The upper bounds of both are 1000 mmol/gDW/h. The requirements are that at least 99.9 % of both rates can be realized by the subnetwork. The original network consists of 526 non-blocked reactions. All the requirements can be fulfilled by using 383 reactions. The number of essential reactions for having at least 20% of the biomass reaction is 369.

***H. pylori* 26695**: One requirement is that at least 99.9 % of the maximal growth rate (20.2606 h^{-1}) can be achieved. Additionally we defined 28 protected reactions. Per default the lower bound of oxygen is -1000 mmol/gDW/h and the one of glucose is 0. The upper bounds of both are 1000 mmol/gDW/h. Protected reactions for the network ***H. pylori* 26695**:

1. Aconitase
2. AKO
3. ATP synthase (four protons for one ATP)
4. BC10 new
5. Citrate synthase
6. CYOO HP
7. Enolase
8. FADOX
9. Fructose-bisphosphate aldolase
10. Fructose-bisphosphatase
11. NAD(P)H-flavin oxidoreductase
12. FRD5
13. FRDO
14. Fumarase
15. Glyceraldehyde-3-phosphate dehydrogenase
16. Isocitrate dehydrogenase (NADP)
17. LDH D1
18. Malate synthase
19. MDH4
20. NADPHQR
21. NDH 1
22. 2-oxoglutarate synthase (rev)
23. PDH2

24. Glucose-6-phosphate isomerase
25. Phosphoglycerate kinase
26. Phosphoglycerate mutase
27. Phosphoenolpyruvate synthase
28. Triose-phosphate isomerase

Reactions which were in all minimal subnetworks in ***H. pylori* 26695**:

1. 1-deoxy-D-xylulose 5-phosphate synthase
2. 1-deoxy-D-xylulose reductoisomerase
3. 1-hydroxy-2-methyl-2-(E)-butenyl 4-diphosphate reductase (dmpp)
4. 1-hydroxy-2-methyl-2-(E)-butenyl 4-diphosphate reductase (ipdp)
5. 2,3-diketo-5-methylthio-1-phosphopentane degradation reaction
6. 2-C-methyl-D-erythritol 2,4-cyclodiphosphate synthase
7. 2-C-methyl-D-erythritol 4-phosphate cytidylyltransferase
8. 2-dehydropantoate 2-reductase
9. 2-oxoglutarate decarboxylase
10. 2-oxoglutarate synthase (rev)
11. 2-succinyl-6-hydroxy-2,4-cyclohexadiene 1-carboxylate synthase
12. 3,4-Dihydroxy-2-butanone-4-phosphate synthase
13. 3-dehydroquinate dehydratase, irreversible
14. 3-dehydroquinate synthase
15. 3-deoxy -D-manno-octulosonic -acid 8-phosphate synthase
16. 3-deoxy-D-arabino-heptulosonate 7-phosphate synthetase
17. 3-deoxy-manno-octulosonate-8-phosphatase
18. 3-methyl-2-oxobutanoate hydroxymethyltransferase
19. 3-phosphoshikimate 1-carboxyvinyltransferase
20. 4,5-dihydroxy-2,3-pentanedione cyclization (spontaneous)
21. 4-(cytidine 5-diphospho)-2-C-methyl-D-erythritol kinase
22. 4-aminobenzoate synthase

23. 5,10-methylenetetrahydrofolate reductase (NADH)
24. 5-Methylthio-5-deoxy-D-ribulose 1-phosphate dehydratase
25. 5-amino-6-(5-phosphoribosylamino)uracil reductase
26. 5-methylthioribose kinase
27. 5-methylthioribose-1-phosphate isomerase
28. 6-carboxyhexanoate-CoA ligase
29. 8-amino-7-oxononanoate synthase
30. ADP-D-glycero-D-manno-heptose epimerase
31. AKO
32. AMMQT6
33. ASPO2
34. ATP synthase (four protons for one ATP)
35. Acetate exchange
36. Acetate kinase
37. Acetate reversible transport via proton symport
38. Acetyl-CoA carboxylase
39. Aconitase
40. Adenine phosphoribosyltransferase
41. Adenosylmethionine decarboxylase
42. Adenosylmethionine-8-amino-7-oxononanoate transaminase
43. Adenylate kinase
44. Adenylosuccinate lyase
45. Adenylosuccinate synthase
46. Adenylsuccinate lyase
47. Agmatinase
48. Alanine racemase
49. Ammonia exchange
50. Ammonia reversible transport

51. Anthranilate phosphoribosyltransferase
52. Arabinose-5-phosphate isomerase
53. Arginase
54. Arginine decarboxylase
55. Asparagine synthetase
56. Aspartate 1-decarboxylase
57. Aspartate carbamoyltransferase
58. Aspartate kinase
59. Aspartate transaminase
60. Aspartate-semialdehyde dehydrogenase
61. BC10 new
62. BTS2
63. Beta-ketoacyl-ACP synthase (2)
64. BiomassHP published
65. C140SN
66. C160SN
67. C180SN
68. C181SN
69. C190cSN
70. CLPNS HP
71. CO2 exchange
72. CO2 transporter via diffusion
73. CTP synthase NH3
74. CYOO HP
75. Carbamoyl-phosphate synthase (glutamine-hydrolysing)
76. Chorismate mutase
77. Chorismate synthase
78. Citrate synthase

79. Cysteine synthase
80. Cytidylate kinase (CMP)
81. D-alanine-D-alanine ligase (reversible)
82. D-glycero-D-manno-hepose 1-phosphate adenyltransferase
83. D-glycero-D-manno-heptose 1,7-bisphosphate phosphatase
84. D-glycero-D-manno-heptose 7-phosphate kinase
85. DASYN HP
86. DHNAOT2
87. DHNPA
88. DHORD3
89. DHPS
90. DTMP kinase
91. Dephospho-CoA kinase
92. Dethiobiotin synthase
93. Diaminohydroxyphosphoribosylaminopyrimidine deaminase
94. Diaminopimelate decarboxylase
95. Diaminopimelate epimerase
96. Dihydrodipicolinate reductase (NADPH)
97. Dihydrodipicolinate synthase
98. Dihydrofolate reductase
99. Dihydrofolate synthase
100. Dihydroneopterin monophosphate dephosphorylase
101. Dihydroneopterin triphosphate pyrophosphatase
102. Dihydroorotase
103. Dimethylallyltranstransferase
104. EX pime LPAREN e RPAREN
105. Enolase
106. FADOX

107. FMN adenylyltransferase
108. FRD5
109. FRDO
110. Formate exchange
111. Formate transport via diffusion
112. Fructose-bisphosphatase
113. Fructose-bisphosphate aldolase
114. Fumarase
115. GDP-D-mannose dehydratase
116. GFUCS
117. GLYCTO1
118. GMP synthase
119. GTP cyclohydrolase I
120. GTP cyclohydrolase II
121. Geranyltranstransferase
122. Glucosamine-1-phosphate N-acetyltransferase
123. Glucose-6-phosphate isomerase
124. Glutamate dehydrogenase (NADP)
125. Glutamate racemase
126. Glutamine phosphoribosyldiphosphate amidotransferase
127. Glutamine synthetase
128. Glutamine-fructose-6-phosphate transaminase
129. Glyceraldehyde-3-phosphate dehydrogenase
130. Glycerol kinase
131. Glycine hydroxymethyltransferase, reversible
132. Glycolaldehyde dehydrogenase
133. Guanylate kinase (GMP:ATP)
134. H+ exchange

135. HCO3 equilibration reaction

136. Heme transport via ABC system

137. Homoserine O trans acetylase

138. Homoserine dehydrogenase (NADPH)

139. Homoserine kinase

140. IMP cyclohydrolase

141. IMP dehydrogenase

142. Indole-3-glycerol-phosphate synthase

143. Inorganic diphosphatase

144. Isochorismate synthase

145. Isocitrate dehydrogenase (NADP)

146. KAS HP

147. KAS HP2

148. KDOCT

149. L arganine reversible transport via proton symport

150. L lactate reversible transport via proton symport

151. L-Arginine exchange

152. L-Histidine exchange

153. L-Isoleucine exchange

154. L-Lactate exchange

155. L-Leucine exchange

156. L-Methionine exchange

157. L-Valine exchange

158. L-glutamate 5-semialdehyde dehydratase (spontaneous)

159. L-histidine transport via ABC system

160. L-isoleucine transport via ABC system

161. L-leucine transport via ABC system

162. L-methionine transport via ABC system

163. L-valine transport via ABC system
164. LDH D1
165. LPADSS HP
166. LPSSYN HP
167. LacR
168. MDH4
169. MECDPDH
170. MOAT HP
171. Malate synthase
172. Malic enzyme (NAD)
173. Malonyl-CoA-ACP transacylase
174. Mannose-1-phosphate guanylyltransferase (GDP)
175. Mannose-6-phosphate isomerase
176. Metb1
177. Methenyltetrahydrofolate cyclohydrolase
178. Methionine adenosyltransferase
179. Methylenetetrahydrofolate dehydrogenase (NADP)
180. Methylthioadenosine nucleosidase
181. NAD kinase
182. NAD synthase (nh3)
183. NAD(P)H-flavin oxidoreductase
184. NADPHQR
185. NDH 1
186. Naphthoate synthase
187. Nicotinate-nucleotide adenylyltransferase
188. Nicotinate-nucleotide diphosphorylase (carboxylating)
189. Nucleoside-diphosphate kinase (ATP:CDP)
190. Nucleoside-diphosphate kinase (ATP:GDP)

191. Nucleoside-diphosphate kinase (ATP:UDP)
192. Nucleoside-diphosphate kinase (ATP:dADP)
193. Nucleoside-diphosphate kinase (ATP:dCDP)
194. Nucleoside-diphosphate kinase (ATP:dGDP)
195. Nucleoside-diphosphate kinase (ATP:dTDP)
196. O-succinylbenzoate-CoA ligase
197. O-succinylbenzoate-CoA synthase
198. O-succinylhomoserine lyase (L-cysteine)
199. O2 exchange
200. O2 transport diffusion
201. Octaprenyl pyrophosphate synthase
202. Ornithine transaminase
203. Orotate phosphoribosyltransferase
204. Orotidine-5-phosphate decarboxylase
205. PABB
206. PASYN HP
207. PDH2
208. PGPP HP
209. PGSA HP
210. PIMEtr
211. PPTGS
212. PSD HP
213. PSSA HP
214. Pantetheine-phosphate adenylyltransferase
215. Pantothenate kinase
216. Pantothenate synthase
217. Phenylalanine transaminase
218. Phosphate exchange

219. Phosphate reversible transport via symport
220. Phospho-N-acetylmuramoyl-pentapeptide-transferase (meso-2,6-diaminopimelate)
221. Phosphoenolpyruvate synthase
222. Phosphoglucomutase
223. Phosphoglucosamine mutase
224. Phosphoglycerate dehydrogenase
225. Phosphoglycerate kinase
226. Phosphoglycerate mutase
227. Phosphomannomutase
228. Phosphopantothenate-cysteine ligase
229. Phosphopantothenoylcysteine decarboxylase
230. Phosphoribosylaminoimidazole carboxylase
231. Phosphoribosylaminoimidazole carboxylase (mutase rxn)
232. Phosphoribosylaminoimidazole synthase
233. Phosphoribosylaminoimidazolecarboxamide formyltransferase
234. Phosphoribosylaminoimidazolesuccinocarboxamide synthase
235. Phosphoribosylanthranilate isomerase (irreversible)
236. Phosphoribosylformylglycinamidine synthase
237. Phosphoribosylglycinamide formyltransferase
238. Phosphoribosylglycinamide synthase
239. Phosphoribosylpyrophosphate synthetase
240. Phosphoserine phosphatase (L-serine)
241. Phosphoserine transaminase
242. Phosphotransacetylase
243. Prephenate dehydratase
244. Prephenate dehydrogenase
245. Protoheme exchange
246. Pyrimidine phosphatase

247. Pyrroline-5-carboxylate reductase
248. Quinolinate synthase
249. RFA HP
250. RFAC HP
251. Riboflavin kinase
252. Riboflavin synthase
253. Ribonucleoside-diphosphate reductase (ADP)
254. Ribonucleoside-diphosphate reductase (CDP)
255. Ribonucleoside-diphosphate reductase (GDP)
256. Ribonucleoside-diphosphate reductase (UDP)
257. Ribose-5-phosphate isomerase
258. Ribulose 5-phosphate 3-epimerase
259. S-adenosylhomocysteine nucleosidase
260. S-ribosylhomocysteine cleavage enzyme
261. SHSL2r
262. Sedoheptulose 7-phosphate isomerase
263. Serine O-acetyltransferase
264. Shikimate dehydrogenase
265. Shikimate kinase
266. Sink ahcys(c)
267. Sink needed to allow 4-hydroxy-5-methyl-3(2H)-furanone to leave system
268. Sink needed to allow S-Adenosyl-4-methylthio-2-oxobutanoate to leave system
269. Spermidine synthase
270. Succinate exchange
271. Succinate transport via proton symport
272. Succinyl-diaminopimelate desuccinylase
273. Succinyldiaminopimelate transaminase

274. Superoxide dismutase
275. TMDSf
276. Tetrahydrodipicolinate succinylase
277. Thiamin exchange
278. Thiamine transport via ABC system
279. Thioredoxin reductase (NADPH)
280. Threonine synthase
281. Transaldolase
282. Transketolase
283. Triose-phosphate isomerase
284. Tryptophan synthase (indoleglycerol phosphate)
285. Tyrosine transaminase
286. U23GAAT HP
287. U2GAAT
288. U2GAAT2
289. UAGAAT HP
290. UAGDP2
291. UDP-N-acetylenolpyruvoylglucosamine reductase
292. UDP-N-acetylglucosamine 1-carboxyvinyltransferase
293. UDP-N-acetylglucosamine diphosphorylase
294. UDP-N-acetylglucosamine-N-acetylmuramyl-(pentapeptide)pyrophosphoryl-undecaprenol N-acetylglucosamine t...
295. UDP-N-acetylmuramoyl-L-alanine synthetase
296. UDP-N-acetylmuramoyl-L-alanyl-D-glutamate synthetase
297. UDP-N-acetylmuramoyl-L-alanyl-D-glutamyl-meso-2,6-diaminopimelate synthetase
298. UDP-N-acetylmuramoyl-L-alanyl-D-glutamyl-meso-2,6-diaminopimeloyl-D-alanyl-D-alanine synthetase
299. UDPglucose 4-epimerase

300. UHGADA HP

301. UMP kinase

302. UNK2

303. USHD HP

304. UTP-glucose-1-phosphate uridylyltransferase (irreversible)

305. Undecaprenyl-diphosphatase

306. Urea exchange

307. Urea transport via facilitate diffusion

308. Uridylate kinase (dUMP)

309. Valine transaminase

M. barkeri str. Fusaro: The maximal biomass rate with ammonia uptake is 0.0268 h^{-1}. Without ammonia, the rate is 0.0095 h^{-1}. Per default the lower bound of ammonia is -9999999 mmol/gDW/h and of glucose 0. The upper bounds of both are 9999999 mmol/gDW/h. The requirements are that at least 99.9 % of both rates can be realized by the subnetwork. The original network consists of 484 non-blocked reactions. All the requirements can be fulfilled by using 364 reactions. The number of essential reactions for having at least 20% of the biomass reaction is 289.

***S. aureus* N315**: The maximal biomass rate with glucose uptake is 8.0759 h^{-1}. Without glucose, the rate is 4.8154 h^{-1}. The requirements are that at least 99.9 % of both rates can be realized by the subnetwork. The original network consists of 465 non-blocked reactions. Per default the lower bounds of oxygen and glucose are -1000 mmol/gDW/h. The upper bounds of both are 1000 mmol/gDW/h. All the requirements can be fulfilled by using 122 reactions. The number of essential reactions for having at least 20% of the biomass reaction is 71.

T. maritima MSB8: The maximal biomass rate with H_2O uptake is 0.2284 h^{-1}. Without H_2O, the rate is 0.1043 h^{-1}. Per default the lower bound of H_2O is -1000 mmol/gDW/h and of glucose 0. The upper bounds of both are 1000 mmol/gDW/h. The requirements are that at least 99.9 % of both rates can be realized by the subnetwork. The original network consists of 385 non-blocked reactions. All the requirements can be fulfilled by using 282 reactions. The number of essential reactions for having at least 20% of the biomass reaction is 267.
